# Complete Genome Sequence of the Bacterium *Bacillus circulans* Jordan Strain 32352

**DOI:** 10.1128/genomeA.00289-17

**Published:** 2017-05-11

**Authors:** Dustin R. Middleton, Walter Lorenz, Fikri Y. Avci

**Affiliations:** a Department of Biochemistry and Molecular Biology, Center for Molecular Medicine and Complex Carbohydrate Research Center, University of Georgia, Athens, Georgia, USA; b Institute of Bioinformatics, University of Georgia, Athens, Georgia, USA

## Abstract

Here, we report the complete genome sequence for the *Bacillus circulans* Jordan strain 32352. This species is a soil dwelling bacterium that expresses glycosyl hydrolase enzymes degrading pneumococcal capsular polysaccharides.

## GENOME ANNOUNCEMENT

The soil microbiome is a tremendously diverse microbial community of bacteria and fungi that produce a variety of enzymes and small molecules relevant to human biology (1, 2). In 1930, Avery and Dubos described a soil-dwelling *Bacillus* sp. that produced an enzyme capable of degrading the type III capsular polysaccharide (Pn3P) of *Streptococcus pneumoniae* (3). A few years later, Sickles and Shaw were able to isolate a similar enzyme-producing strain of *Bacillus palustris* (renamed later *Bacillus circulans*) from decaying organic matter in soil. They described this bacterium as a sporulating, Gram-negative, aerobic bacillus with peritrichous flagella (4). In several subsequent studies, researchers have utilized culture filtrate preparations of this Pn3P-degrading enzyme (Pn3Pd) while investigating Pn3P biosynthesis and its antigenic and immunological properties (5 –7). We set out to sequence this bacterium in order to identify the enzyme responsible for Pn3P depolymerization and other potential carbohydrate-active enzymes produced in this strain.

*B. circulans* Jordan strain 32352 was acquired from the American Type Culture Collection (ATCC 14175). Genomic DNA was isolated using the DNeasy blood and tissue kit (Qiagen, USA). Genomic DNA was submitted to the Georgia Genomics Facility (University of Georgia) for DNA library synthesis using a KAPA Hyper kit (Kapa Biosystems, USA) and TrueSeq LT adapters. Paired-end (PE) 150-bp reads were sequenced on the Illumina NextSeq 500 system (Illumina, Inc., USA) with NextSeq version 2 reagents.

Genome assembly and analysis was performed by the Quantitative Biology Consulting Group (University of Georgia). Raw and trimmed reads were assessed using FastQC (8) and quality trimming was done using Trimmomatic (9) with the following settings: ILLUMINACLIP:TruSeq3-PE-2.fa:2:30:10, LEADING:20, TRAILING:15, SLIDINGWINDOW:4:25, and MINLEN:50. Genome assembly was performed using SPAdes version 3.9 software (10) with both paired and unpaired reads as inputs. Assembly metrics were determined using QUAST (11), and genome annotation was performed on the RAST server (12). Identification of putative prophage elements was done with PHAST (13).

Trimmed reads (ca. 2.4 million paired and 1.54 million unpaired) representing 150× base coverage were assembled. The genome assembly of *B. circulans* Jordan strain 32352 contained 494 contigs, of which 40 were ≥500 bp. The largest scaffold was 1.36 Mb in length, and the total assembly length was 7.92 Mb with an *N*_50_ of 433 kb and an *L*_50_ of 6. The GC content was 49.3%. RAST annotation predicted 7,269 coding sequences, and PHAST analysis detected 2 regions containing questionable and incomplete prophage elements, respectively.

### Accession number(s).

This whole-genome shotgun project has been deposited at DDBJ/ENA/GenBank under the accession number MZNT00000000. The version described in this paper is the first version, MZNT01000000.
